# What Controls Variation in Human Skin Color?

**DOI:** 10.1371/journal.pbio.0000027

**Published:** 2003-10-13

**Authors:** Gregory S Barsh

## Abstract

There is a large range of human skin color, yet we know very little about the underlying genetic architecture. Is the number of skin color genes close to five, 50, or 500?

Diversity of human appearance and form has intrigued biologists for centuries, but nearly 100 years after the term “genetics” was coined by William Bateson in 1906, the genes that underlie this diversity are an unsolved mystery. One of the most obvious phenotypes that distinguish members of our species, differences in skin pigmentation, is also one of the most enigmatic. There is a tremendous range of human skin color in which variation can be correlated with climates, continents, and/or cultures, yet we know very little about the underlying genetic architecture. Is the number of common skin color genes closer to five, 50, or 500? Do gain- and loss-of-function alleles for a small set of genes give rise to phenotypes at opposite ends of the pigmentary spectrum? Has the effect of natural selection on similar pigmentation phenotypes proceeded independently via similar pathways? And, finally, should we care about the genetics of human pigmentation if it is only skin-deep?

## Why Should We Care?

From a clinical perspective, inadequate protection from sunlight has a major impact on human health (Armstrong et al. 1997; Diepgen and Mahler 2002). In Australia, the lifetime cumulative incidence of skin cancer approaches 50%, yet the oxymoronic “smart tanning” industry continues to grow, and there is controversy over the extent to which different types of melanin can influence susceptibility to ultraviolet (UV) radiation (Schmitz et al. 1995; Wenczl et al. 1998). At the other end of the spectrum, inadequate exposure to sunlight, leading to vitamin D deficiency and rickets, has been mostly cured by nutritional advances made in the early 1900s. In both cases, understanding the genetic architecture of human skin color is likely to provide a greater appreciation of underlying biological mechanisms, much in the same way that mutational hotspots in the gene *TP53* have helped to educate society about the risks of tobacco (Takahashi et al. 1989; Toyooka et al. 2003).

From a basic science perspective, variation in human skin color represents an unparalleled opportunity for cell biologists, geneticists, and anthropologists to learn more about the biogenesis and movement of subcellular organelles, to better characterize the relationship between genotypic and phenotypic diversity, to further investigate human origins, and to understand how recent human evolution may have been shaped by natural selection.

## The Color Variation Toolbox

**Figure 1:**
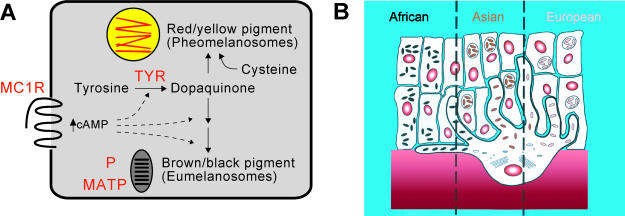
Biochemistry and Histology of Different Skin Types (A) Activation of the melanocortin 1 receptor (MC1R) promotes the synthesis of eumelanin at the expense of pheomelanin, although oxidation of tyrosine by tyrosinase (TYR) is required for synthesis of both pigment types. The membrane-associated transport protein (MATP) and the pink-eyed dilution protein (P) are melanosomal membrane components that contribute to the extent of pigment synthesis within melanosomes. (B) There is a gradient of melanosome size and number in dark, intermediate, and light skin; in addition, melanosomes of dark skin are more widely dispersed. This diagram is based on one published by Sturm et al. (1998) and summarizes data from Szabo et al. (1969), Toda et al. (1972), and Konrad and Wolff (1973) based on individuals whose recent ancestors were from Africa, Asia, or Europe.

Historically, measurement of human skin color is often based on subjective categories, e.g., “moderate brown, rarely burns, tans very easily.” More recently, quantitative methods based on reflectance spectrophotometry have been applied, which allow reddening caused by inflammation and increased hemoglobin to be distinguished from darkening caused by increased melanin (Alaluf et al. 2002b; Shriver and Parra 2000; Wagner et al. 2002). Melanin itself is an organic polymer built from oxidative tyrosine derivatives and comes in two types, a cysteine-rich red–yellow form known as pheomelanin and a less-soluble black--brown form known as eumelanin (Figure 1A). Discriminating among pigment types in biological samples requires chemical extraction, but is worth the effort, since the little we do know about common variation in human pigmentation involves pigment type-switching. The characteristic phenotype of fair skin, freckling, and carrot-red hair is associated with large amounts of pheomelanin and small amounts of eumelanin and is caused by loss-of-function alleles in a single gene, the melanocortin 1 receptor *(MC1R)* (Sturm et al. 1998; Rees 2000) However, *MC1R* variation has a significant effect on pigmentation only in populations where red hair and fair skin are common (Rana et al. 1999; Harding et al. 2000), and its primary effects—to promote eumelanin synthesis at the expense of pheomelanin synthesis, or vice versa— contribute little to variation of skin reflectance among or between major ethnic groups (Alaluf et al. 2002a).

More important than the ratio of melanin types is the total amount of melanin produced. In addition, histological characteristics of different-colored skin provide some clues as to cellular mechanisms that are likely to drive pigmentary variation (Figure 1B). For the same body region, light- and dark-skinned individuals have similar numbers of melanocytes (there is considerable variation between different body regions), but pigment-containing organelles, called melanosomes, are larger, more numerous, and more pigmented in dark compared to intermediate compared to light skin, corresponding to individuals whose recent ancestors were from Africa, Asia, or Europe, respectively (Szabo et al. 1969; Toda et al. 1972; Konrad and Wolff 1973). From these perspectives, oxidative enzymes like tyrosinase (TYR), which catalyzes the formation of dopaquinone from tyrosine, or melanosomal membrane components like the pink-eyed dilution protein (P) or the membrane-associated transporter protein (MATP), which affect substrate availability and activity of TYR (Orlow and Brilliant 1999; Brilliant and Gardner 2001; Newton et al. 2001; Costin et al. 2003), are logical candidates upon which genetic variation could contribute to the diversity of human skin color.

Of equal importance to what happens inside melanocytes is what happens outside. Each pigment cell actively transfers its melanosomes to about 40 basal keratinocytes; ultimately, skin reflectance is determined by the amount and distribution of pigment granules within keratinocytes rather than melanocytes. In general, melanosomes of African skin are larger and dispersed more widely than in Asian or European skin (Figure 1). Remarkably, keratinocytes from dark skin cocultured with melanocytes from light skin give rise to a melanosome distribution pattern characteristic of dark skin, and vice versa (Minwalla et al. 2001). Thus, at least one component of skin color variation represents a gene or genes whose expression and action affect the pigment cell environment rather than the pigment cell itself.

## Genetics of Skin Color

For any quantitative trait with multiple contributing factors, the most important questions are the overall heritability, the number of genes likely to be involved, and the best strategies for identifying those genes. For skin color, the broad sense heritability (defined as the overall effect of genetic vs. nongenetic factors) is very high (Clark et al. 1981), provided one is able to control for the most important nongenetic factor, exposure to sunlight.

Statements regarding the number of human skin color genes are attributed to several studies; one of the most complete is by Harrison and Owen (1964). In that study, skin reflectance measurements were obtained from 70 residents of Liverpool whose parents, grandparents, or both were of European (“with a large Irish component”) or West African (“mostly from coastal regions of Ghana and Nigeria”) descent and who were roughly classified into “hybrid” and “backcross” groups on this basis. An attempt to partition and analyze the variance of the backcross groups led to minimal estimates of three to four “effective factors,” in this case, independently segregating genes. Aside from the key word *minimal* (Harrison and Owen's data could also be explained by 30–40 genes), one of the more interesting findings was that skin reflectance appeared to be mainly additive. In other words, mean skin reflectance of “F1 hybrid” or “backcross hybrid” groups is intermediate between their respective parental groups.

An alternative approach for considering the number of potential human pigmentation genes is based on mouse coat color genetics, one of the original models to define and study gene action and interaction, for which nearly 100 different genes have been recognized (Bennett and Lamoreux 2003; Jackson 1994). Setting aside mouse mutations that cause white spotting or predominant effects outside the pigmentary system, no more than 15 or 20 mutations remain, many of which have been identified and characterized, and most of which have human homologs in which null mutations cause albinism.

This brings us to the question of candidate genes for skin color, since, like any quantitative trait, a reasonable place to start is with rare mutations known to cause an extreme phenotype, in this case Mendelian forms of albinism. The underlying assumption is that if a rare null allele causes a complete loss of pigment, then a set of polymorphic, i.e., more frequent, alleles with subtle effects on gene expression will contribute to a spectrum of skin colors. The *TYR, P*, and *MATP* genes discussed earlier are well-known causes of albinism whose primary effects are limited to pigment cells (Oetting and King 1999); among these, the *P* gene is highly polymorphic but the phenotypic consequences of *P* gene polymorphisms are not yet known.

Independent of phenotype, a gene responsible for selection of different skin colors should exhibit a population signature with a large number of alleles and rates of sequence substitution that are greater for nonsynonymous (which change an amino acid in the protein) than synonymous (which do not change any amino acid) alterations. Data have been collected only for *MC1R*, in which the most notable finding is a dearth of allelic diversity in African samples, which is remarkable given that polymorphism for most genes is greater in Africa than in other geographic regions (Rana et al. 1999; Harding et al. 2000). Thus, while *MC1R* sequence variation does not contribute significantly to variation in human skin color around the world, a functional *MC1R* is probably important for dark skin.

## Selection for Skin Color?

**Figure 2:**
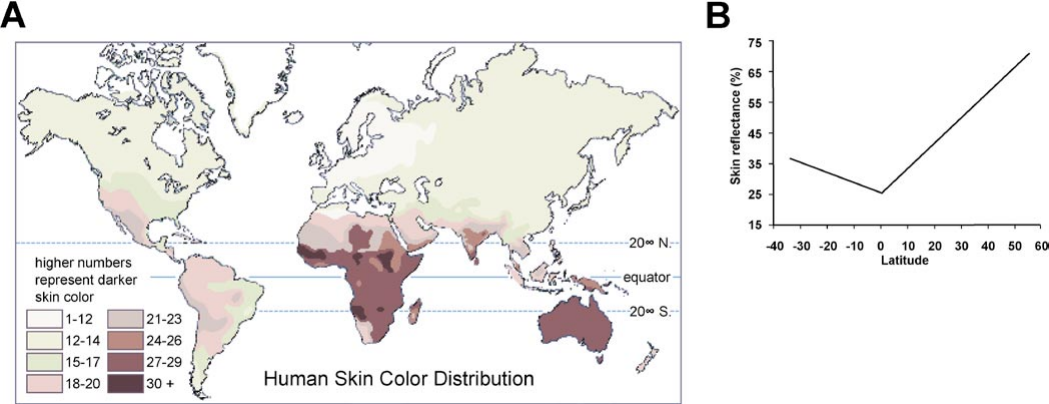
Relationship of Skin Color to Latitude (A) A traditional skin color map based on the data of Biasutti. Reproduced from http://anthro.palomar.edu/vary/ with permission from Dennis O'Neil.
Erratum note: The source of this image was incorrectly acknowledged. Corrected 12/19/03.
(B) Summary of 102 skin reflectance samples for males as a function of latitude, redrawn from Relethford (1997).

Credit for describing the relationship between latitude and skin color in modern humans is usually ascribed to an Italian geographer, Renato Basutti, whose widely reproduced “skin color maps” illustrate the correlation of darker skin with equatorial proximity (Figure 2). More recent studies by physical anthropologists have substantiated and extended these observations; a recent review and analysis of data from more than 100 populations (Relethford 1997) found that skin reflectance is lowest at the equator, then gradually increases, about 8% per 10° of latitude in the Northern Hemisphere and about 4% per 10° of latitude in the Southern Hemisphere. This pattern is inversely correlated with levels of UV irradiation, which are greater in the Southern than in the Northern Hemisphere. An important caveat is that we do not know how patterns of UV irradiation have changed over time; more importantly, we do not know when skin color is likely to have evolved, with multiple migrations out of Africa and extensive genetic interchange over the last 500,000 years (Templeton 2002).

Regardless, most anthropologists accept the notion that differences in UV irradiation have driven selection for dark human skin at the equator and for light human skin at greater latitudes. What remains controversial are the exact mechanisms of selection. The most popular theory posits that protection offered by dark skin from UV irradiation becomes a liability in more polar latitudes due to vitamin D deficiency (Murray 1934). UVB (short-wavelength UV) converts 7-dehydrocholesterol into an essential precursor of cholecaliferol (vitamin D_3_); when not otherwise provided by dietary supplements, deficiency for vitamin D causes rickets, a characteristic pattern of growth abnormalities and bony deformities. An oft-cited anecdote in support of the vitamin D hypothesis is that Arctic populations whose skin is relatively dark given their latitude, such as the Inuit and the Lapp, have had a diet that is historically rich in vitamin D. Sensitivity of modern humans to vitamin D deficiency is evident from the widespread occurrence of rickets in 19th-century industrial Europe, but whether dark-skinned humans migrating to polar latitudes tens or hundreds of thousands of years ago experienced similar problems is open to question. In any case, a risk for vitamin D deficiency can only explain selection for light skin. Among several mechanisms suggested to provide a selective advantage for dark skin in conditions of high UV irradiation (Loomis 1967; Robins 1991; Jablonski and Chaplin 2000), the most tenable are protection from sunburn and skin cancer due to the physical barrier imposed by epidermal melanin.

## Solving the Mystery

Recent developments in several areas provide a tremendous opportunity to better understand the diversity of human pigmentation. Improved spectrophotometric tools, advances in epidemiology and statistics, a wealth of genome sequences, and efficient techniques for assaying sequence variation offer the chance to replace misunderstanding and myths about skin color with education and scientific insight. The same approaches used to investigate traits such as hypertension and obesity—genetic linkage and association studies—can be applied in a more powerful way to study human pigmentation, since the sources of environmental variation can be controlled and we have a deeper knowledge of the underlying biochemistry and cell biology.

This approach is especially appealing given the dismal success rate in molecular identification of complex genetic diseases. In fact, understanding more about the genetic architecture of skin color may prove helpful in designing studies to investigate other quantitative traits. Current debates in the human genetics community involve strategies for selecting populations and candidate genes to study, the characteristics of sequence polymorphisms worth pursuing as potential disease mutations, and the extent to which common diseases are caused by common (and presumably ancient) alleles. While specific answers will be different for every phenotype, there may be common themes, and some answers are better than none.

Harrison and Owen concluded their 1964 study of human skin color by stating, “The deficiencies in the data in this study are keenly appreciated by the writers, but since there appear at present to be no opportunities for improving the data, it seems justifiable to take the analysis as far as possible.” Nearly 40 years later, opportunities abound, and the mystery of human skin color is ready to be solved. 
